# IQGAP1 control of centrosome function defines distinct variants of triple negative breast cancer

**DOI:** 10.18632/oncotarget.27623

**Published:** 2020-06-30

**Authors:** Mahasin A. Osman, William James Antonisamy, Evgeny Yakirevich

**Affiliations:** ^1^ Department of Medicine, Division of Oncology, Health Sciences Campus, University of Toledo, Toledo, OH 43614, USA; ^2^ Department of Molecular Pharmacology, Physiology and Biotechnology, Division of Biology and Medicine, Warren Alpert Medical School of Brown University, Providence, RI 02912, USA; ^3^ Department of Pathology and Laboratory Medicine, Rhode Island Hospital, Warren Alpert Medical School of Brown University, Providence, RI 02903, USA

**Keywords:** IQGAP1, BRCA1, β-catenin, MNK1, triple negative breast cancer

## Abstract

Triple negative breast cancer (TNBC) is a heterogenous and lethal disease that lacks diagnostic markers and therapeutic targets; as such common targets are highly sought after. IQGAP1 is a signaling scaffold implicated in TNBC, but its mechanism is unknown. Here we show that IQGAP1 localizes to the centrosome, interacts with and influences the expression level and localization of key centrosome proteins like BRCA1 and thereby impacts centrosome number. Genetic mutant analyses suggest that phosphorylation cycling of IQGAP1 is important to its subcellular localization and centrosome-nuclear shuttling of BRCA1; dysfunction of this process defines two alternate mechanisms associated with cell proliferation. TNBC cell lines and patient tumor tissues differentially phenocopy these mechanisms supporting clinical existence of molecularly distinct variants of TNBC defined by IQGAP1 pathways. These variants are defined, at least in part, by differential mis-localization or stabilization of IQGAP1-BRCA1 and rewiring of a novel Erk1/2-MNK1-JNK-Akt-β-catenin signaling signature. We discuss a model in which IQGAP1 modulates centrosome-nuclear crosstalk to regulate cell division and imparts on cancer. These findings have implications on cancer racial disparities and can provide molecular tools for classification of TNBC, presenting IQGAP1 as a common target amenable to personalized medicine.

## INTRODUCTION

The triple negative breast cancer (TNBC) is a highly heterogeneous group of diseases defined by absence of expression of growth factor and hormonal receptors, and thus it is highly lethal due to lack of diagnostic markers and therapeutic targets [1–4]. Familial TNBC has been defined by mutations in the tumor suppressor gene, breast cancer-associated 1, *BRCA1* [5], however, the origin of sporadic TNBC remains obscure [6]. Dysfunction of wild type BRCA1 protein also associates with cancer [7–10], but its mechanism is unclear. BRCA1 has diverse cellular functions, including mitosis that has been linked to its interaction with the centrosome markers γ-tubulin and pericentrin to regulate centrosome number [11, 12]. In vitro depletion of BRCA1 results in amplified centrosomes [12–14], a phenotype observed in early-stage tumors, including breast cancer [15, 16], but how might wild type BRCA1 protein control centrosome amplification is unclear.

Aberrant activity of the IQ-containing GTPase Activating Protein (IQGAP1) associates with many carcinomas, including TNBC [17–19]. While overexpression of IQGAP1 has been implicated in these carcinomas and proposed as clinical target [19–21], its mechanism is just emerging. IQGAP1 is a regulatory scaffold with remarkable signaling versatility stemming from its ability to assemble signaling sub-complexes that respond to various stimuli and generate highly specific cellular responses by selecting the appropriate downstream targets in a context-dependent manner [19, 22, 23]. IQGAP1 modulates oncogenic pathways like mTOR-S6K-Akt pathway and the mitogen protein kinase (MAPK) Erk1/2 [23, 24], and controls adheren and tight junctions in epithelial cells by regulating the E-cadherin-β-catenin complex [25, 26]. Importantly, IQGAP1 plays an essential role in mitosis [27], localizing with centrosomal markers in mid-body ring during cell abscission [24]. Furthermore, proteomic analyses identified IQGAP1 among centrosome-bound proteins implicated in cell abscission [28]. However, the role of IQGAP1 in centrosome function is unknown.

In animal cells, the centrosome is the microtubule organizing center (MTOC) that generates cytoskeleton, aster and the spindle microtubules, which segregate the chromosomes to daughter cells during mitosis [29, 30]. Beside their role in cytoskeleton organization, microtubules serve as a signal transduction platform during cell division and has long been target of cancer therapy [31]. The centrosome contains two centrioles surrounded by pericentriolar material (PCM) and a number of various proteins some of which serve as centrosome-specific markers [32]. Specifically, acetylation of α-tubulin on lysine 40 (K40) is a well-known marker of stabilized microtubules [33], and has been implicated in the metastatic potential of breast cancer [34]. On the other hand, increased expression or delocalization of γ-tubulin from the centrosome to the cytoplasm has been observed in breast cancer cell lines [31, 35]. Another important centrosome/centriole marker is the resident protein centrin that plays fundamental roles in centrosome structure and function such as centriole duplication and regulation of cytokinesis [36].

The centrosome divides only once per cell cycle to deliver the proper number of chromosomes to each daughter cell [30]. Centrosome aberrations widely associate with human malignancies and are a candidate hallmark of cancer [37, 38]. While increased centrosome size resulting from PCM expansion has been reported as abnormality in human tumors [39], increased centrosome number is observed in 20–30% of tumors that overexpress oncogenes or lack tumor suppressors like BRCA1 [40, 41]. Centrosome amplification has been associated with high-grade tumors and poor prognosis and was suggested as a biomarker for advanced cancer [37, 42]. More recent evidence strongly supports that centrosome amplification represents an earlier step in tumorigenesis and contributes to tumor metastasis [43]. However, the mechanisms underlying centrosome aberrations remain incompletely understood [30].

In this study, we present a novel mechanism for IQGAP1 in tumorigenesis associated with centrosome aberrations. We report that IQGAP1 interacts with centrosome proteins and influences their expression level and subcellular localization. Expression of dominant active mutants of IQGAP1 associates with amplified centrosomes while expression of dominant negative mutants associates with increased centrosome size. IQGAP1 binds BRCA1 and influences its subcellular distribution, and affects the expression level of the key centrosome markers centrin, acetylated α-tubulin and γ-tubulin. These phenotypes differentially associate with TNBC cell lines, activate specific IQGAP1-signaling signatures, and they have clinical significance, as they similarly associate with human TNBC tumors. We discuss a model whereby IQGAP1 acts as a signaling scaffold in the centrosome and influences centrosome protein transport, dysfunction of which underlie centrosome aberrations in cancer thereby presenting IQGAP1 as a common target in variants of TNBC, amenable to personalized medicine.

## RESULTS

### IQGAP1 localizes to the centrosome and impacts centrosome size and number

**Figure 1:**
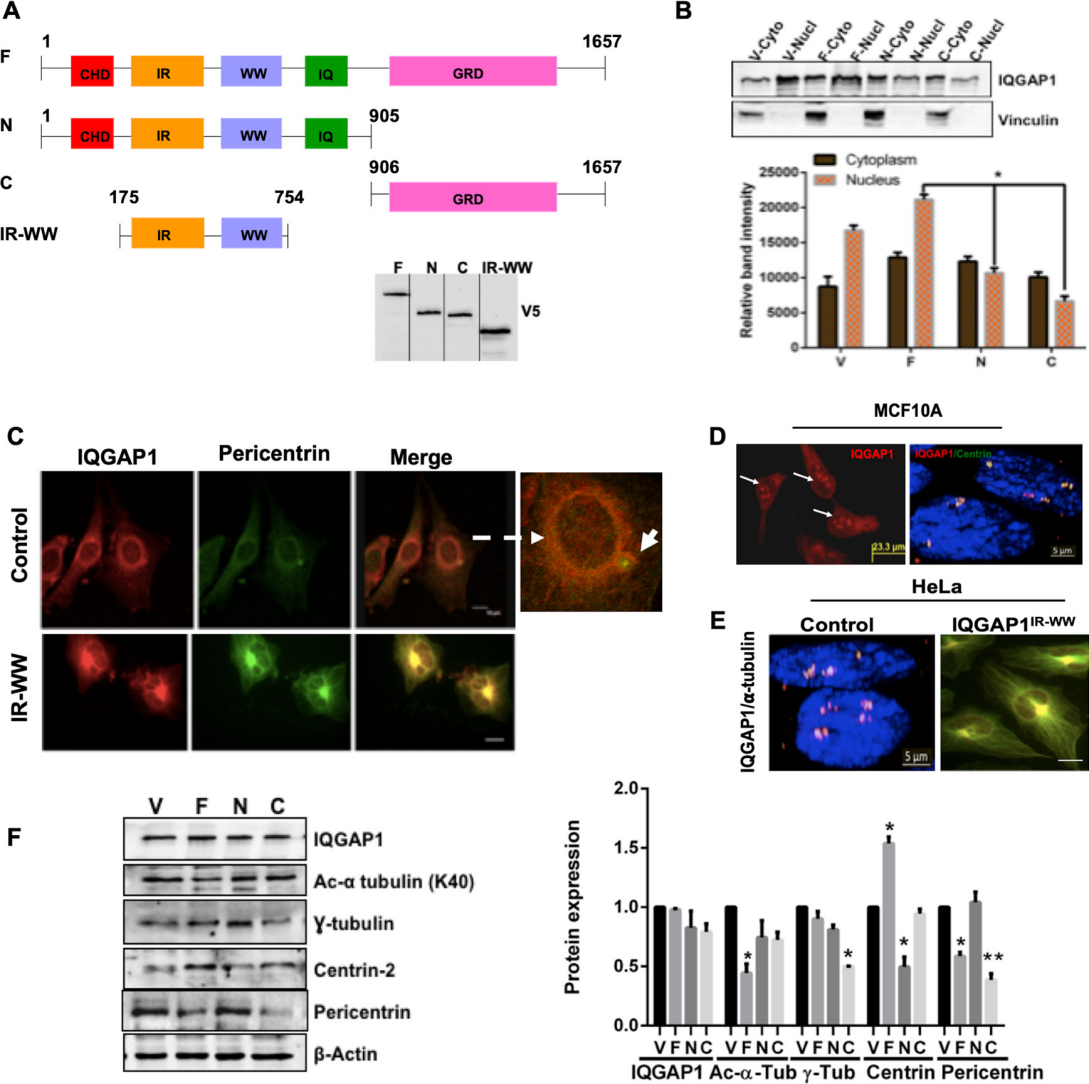
IQGAP1 localizes to the centrosome and modulates centrosome size and number. (**A**) Schematic domain structure of human IQGAP1 and constructs used in this study: CHD; *c*alponin *h*omology *d*omain; IR-WW: *I*QGAP1-*r*epeats (IR) and tryptophan (WW) repeats; IQ: four *i*soleucine and *g*lutamine rich motifs; GRD: *R*asGTPase-activating protein-*r*elated *d*omain where the critical Ser 1443, the NLS: nuclear localization signal and the aPI motif that binds phospho-lipid PIP3 are located. Microtubule and β-catenin binding sites are also located in the far C-terminus. F: IQGAP1-full length whose expression serves as dominant active. N: IQGAP1 N-terminus half, and IR-WW refer to IQGAP1-N and IQGAP1^IR-WW^ truncations respectively both act as dominant negative. C: is IQGAP1 C-terminus half, IQGAP1-C, whose expression serves as dominant active. The mutants act by binding to and altering endogenous IQGAP1 localization and activity (phosphorylation status). Western blot shows stable expression of the V5-tagged constructs in MCF10A cells as blotted by V5 antibodies. (**B**) Expression of IQGAP1 Dominant Mutants Alters its Subcellular Distribution: Extracts from MCF10A cells stably expressing empty vector control (V), IQGAP1-F (F), IQGAP-N (N) or IQGAP1-C (C) were fractionated and evaluated by Western blot (upper panel) for endogenous iQGAP1 and the band intensities quantified by densitometry (lower panel). Error bars are Means± standard deviation (s. d) for 3 independent experiments (*n =* 3). (**C**) IQGAP1 localizes to the centrosome: MCF10A were co-stained for endogenous IQGAP1 (red) and the centrosome marker pericentrin (green, arrow) and visualized by confocal microscopy (Upper panels). MCF10A cells stably expressing V5-IQGAP1^IR-WW^ were co-stained with antibodies for V5 (red) and endogenous pericentrin (green) where they co-localize to an enlarged centrosome (lower panels). Scale bars = 10 mm. (**D**) Expression of IQGAP1-F increases centrosome number: MCF10A stably expressing V5-IQGAP1-F were co-stained with antibodies for V5 (red), the centrosome marker centrin (green) and DAPI for nucleus (blue), and visualized by fluorescence microscope for IQGAP1 (left) and by three-channel super-resolution microscopy. Scale bar = 5 mm. (**E**) Expression of the dominant negative IQGAP1^IR-WW^ abolishes amplified centrosomes and produces enlarged centrosome in cancer cells: Control Hela cells with amplified centrosome (left), and Hela cells stably expressing V5-IQGAP1^IR-WW^ were co-stained with antibodies for endogenous IQGAP1 or V5 (red) and the centrosome marker α-tubulin (green) and visualized by confocal microscopy. Scale bar = 5 mm. (**F**) IQGAP1 modulates the expression levels of centrosome markers: Cell lysate extracted from MCF10A cells stably expressing IQGAP1 constructs was evaluated by Western blotting with antibodies for endogenous IQGAP1 and the indicated centrosome markers (left panel) and the band intensities quantified by densitometry (right graph). Error bars are the Means± s. d for *n =* 3, *p-*value: ^*^ 0.05, ^**^0.01.

As shown in Figure 1A, IQGAP1 is a modular protein involved in many cellular functions [17, 19]. Previously, we demonstrated that IQGAP1 acts as a phosphorylation-dependent conformation switch that regulates insulin secretion, cell size and proliferation [24, 27, 44]. Using functional genetic analyses, we created several tagged dominant mutants, by truncation and point mutations in the different domains of IQGAP1. Our analyses established dominant active mutants that inhibit insulin secretion and reduce cell size while accelerating the cell cycle, leading to cell transformation, and dominant negative mutants that increase insulin secretion and cell size while arresting cytokinesis, leading to production of multi-nucleated cells [24, 27]. Whereas the dominant active mutants induce cell transformation by bypassing growth factor signaling, the dominant negative mutants can induce cell transformation in presence of growth factors like EGF [24]. In this study we used V5-tagged IQGAP1-F and IQGAP1-C as facile surrogates of the dominant active mutants that mimic the phosphorylated form of IQGAP1, and IQGAP1-N and IQGAP1^IR-WW^ as surrogates of the dominant negative mutants that mimic the un-phosphorylated form of IQGAP1 (Figure 1) [24, 27, 44].

To further understand the mechanism of IQGAP1 in cell proliferation, we performed cell fractionation assays in the immortalized normal mammary cells MCF10A expressing the dominant mutants. In control MCF10A cells expressing the empty vector, more IQGAP1 was found in the nucleus but the expression of the constructs altered IQGAP1 subcellular distribution (Figure 1B). Expression of IQGAP1-F enhanced nuclear distribution of IQGAP1 whereas expression of IQGAP1-N or IQGAP1-C both reduced the nuclear localization of IQGAP1, and slightly enhanced the cytoplasmic fraction (Figure 1B). These results present new direction supporting our earlier conclusion that negative and active mutants promote cell proliferation by distinct mechanisms [24, 27]. Accordingly, we set out to uncover these mechanisms starting by examining the cells microscopically. Indeed, we observed new localization patterns of IQGAP1 in the different mutant cells.

In control MCF10A, IQGAP1 localized to the nuclear envelope and extended into the centrosome co-localizing with the centrosome marker pericentrin (Figure 1C, upper panels). Expression of the dominant negative mutant IQGAP1^IR-WW^ associated with enlarged centrosome as denoted by co-localization of IQGAP1 with pericentrin on large centrosome structure (Figure 1C lower panel). Multiple nuclei can be seen hooked to that large centrosome structure consistent with our previous report that this mutant arrests cytokinesis, producing multiple nuclei [27]. In contrast, expression of IQGAP1-F and dominant active mutant IQGAP1-C associated with increased number of centrosomes as evident by co-localization of IQGAP1 with another centrosome/centriole marker, centrin on amplified centrosomes (Figure 1D).

To ascertain this observation and substantiate the notion that IQGAP1 influences centrosome number via different domains, we used HeLa cells known for containing supernumerary centrosomes. Expression of IQGAP1^IR-WW^ suppressed the amplified centrosome phenotype in HeLa cells, producing a single enlarged centrosome as evident by co-localization of IQGAP1 with yet another centrosome marker, α-tubulin (Figure 1E).

Next, we asked whether IQGAP1 influences centrosome size and number by affecting the expression levels of centrosome proteins. Quantitative immunoblotting analyses demonstrates that expression of the different IQGAP1 constructs differentially affects the expression level of the known centrosome markers (Figure 1F). The level of acetylated (Ac) α-tubulin was significantly reduced in IQGAP1-F cells, while the level of γ-tubulin was significantly reduced in IQGAP1-C cells. The level of centrin was slightly reduced in IQGAP1-F, but significantly so in IQGAP1-N cells. For interest in understanding mechanism involved in the development of triple negative breast cancer (TNBC), we chose to further examine how IQGAP1 might influence BRCA1 because it is both a centrosome marker and is associated with TNBC.

### IQGAP1 interacts with and influences BRCA1 subcellular distribution

**Figure 2:**
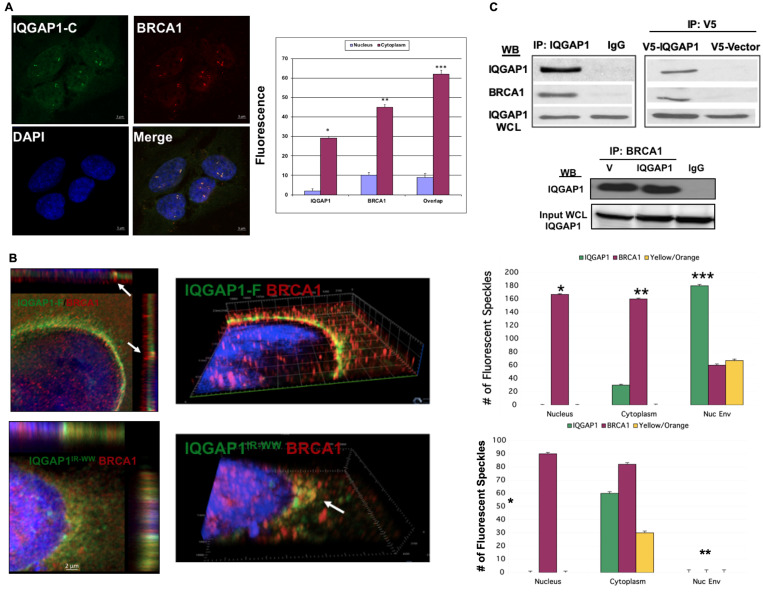
IQGAP1 interacts with and modulates the subcellular distribution of the centrosome marker BRCA1. (**A**) Dominant Active IQGAP1-C fragment promotes centrosome amplification where IQGAP1-BRCA1 co-localize: MCF10A cells stably expressing dominant active IQGAP1-C were co-stained with antibodies for endogenous IQGAP1 (green), BRCA1 (red) and DAPI for nuclei (blue) and visualized by super-resolution microscopy (left). Subcellular distribution of IQGAP1 and BRCA1 was quantified from 50 random cells. Error bars are the Means ± s. d for *n =* 3, *p-*value ^*^ 0.05, ^**^ 0.01. (**B**) Altered subcellular distribution of BRCA1 in IQGAP1 dominant mutants: left panels, MCF10A cells stably expressing IQGAP1-F (upper two panels) or IQGAP1^IR-WW^ (lower two panels) were co-stained for endogenous IQGAP1 and BRCA1 and visualized by super-resolution microscopy. Right panels, the subcellular distribution (nuclear, nuclear envelope, cytoplasm) of IQGAP1 and BRCA1 was quantified from 50 random cells each. Error bars are the Means± s. d for *n =* 3, *p-*value ^*^ 0.05, ^**^ 0.01, ^***^ 0.001. (**C**) IQGAP1 and BRCA1 interact: Upper panels, left. Representative immuno-blot of co-precipitation of BRCA1 with IQGAP1 from total MCF10A cell lysate. IgG are sham IP. Endogenous IQGAP1 in whole cell lysate (WCL) denotes equal input. Upper panels, right: Exogenous IQGAP1 interacts with endogenous BRCA1: V5 antibodies were used to IP V5-IQGAP1-F from lysates. V5 denotes negative control from cells expressing empty V5-vector. Endogenous IQGAP1 blotted from WCL denotes equal input. The blots represent at least three independent experiments. Lower Panels. Reciprocal IP of IQGAP1 with BRCA1: monoclonal antibodies for BRCA1 co-precipitate endogenous IQGAP1 from control cells expressing empty vector (V) or exogenous V5-IQGAP1-F. IgG denotes mock IP as negative control. IQGAP1 in the whole cell lysate (WCL) was blotted to demonstrate equal input.

We examined how IQGAP1 influences BRCA1 using the cell lines stably expressing IQGAP1 mutants (Figure 2). Figure 2A, left panels, shows that in cells stably expressing the dominant active IQGAP1-C, BRCA1 co-localizes with endogenous IQGAP1 on amplified centrosomes. Un-colocalized pools of IQGAP1 and BRCA1 remaining either in the nucleus or cytoplasm were also observed (graph, right). The super-resolution confocal slices in Figure 2B, upper panels, show that BRCA1 also co-localizes with IQGAP1 at interval points in the nuclear envelop in dominant active IQGAP1-F whereas some un-colocalized pools remain inside the nucleus or cytoplasm. This pattern is clearly apparent in the 3D slice in the middle and the quantification graph to the right. In contrast, Figure 2B, lower left panels, show that IQGAP1-BRCA1 co-localization pattern on the nuclear envelope was lost in the dominant negative IQGAP1^IR-WW^ cells. Instead, in these cells, IQGAP1-BRCA1 co-localized in cytoplasmic aggregates near the nucleus (Figure 2B, lower middle). The dramatic co-localization shift from nuclear envelop to cytoplasm aggregates was clearer when comparing the upper and lower graphs in Figure 2B.

The effects of IQGAP1 mutants on BRCA1 localization suggested that IQGAP1 modulates BRCA1 subcellular distribution through physical interaction. This idea was tested by reciprocal immunoprecipitation (IP). Figure 2C, left panel, shows that endogenous IQGAP1 can co-precipitate endogenous BRCA1. Similarly, exogenous V5-IQGAP1 can co-precipitate endogenous BRCA1 (Figure 2B left), and antibodies for BRCA1 can co-precipitate both IQGAP1 and BRCA1 from control or IQGAP1 stable cells (Figure 2C, lower panel). Altogether, these findings suggest that IQGAP1 plays an important role in the centrosome by which it controls cell proliferation and that it may impact cancer development. This idea was tested in TNBC cell lines.

### Differential expression and/or spatial distribution of IQGAP1, BRCA1 and centrosome markers in TNBC Cell Lines

**Figure 3:**
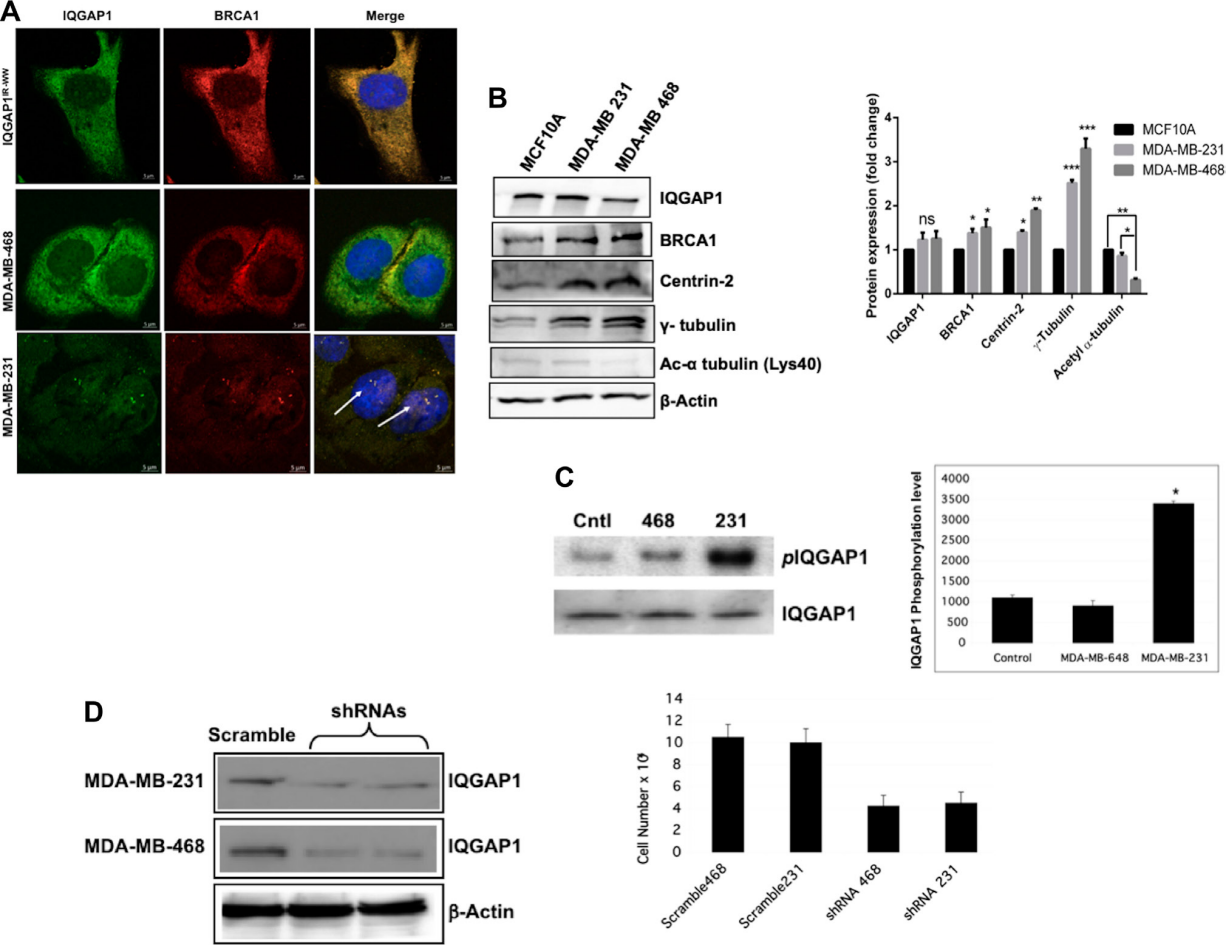
Differential expression and/or spatial distribution of IQGAP1, BRCA1 and centrosome markers in different Triple Negative Breast Cancer (TNBC) cell lines. (**A**) Localization of IQGAP1-BRCA1 in TNBC cell lines. Localization of endogenous IQGAP1 (green) and BRCA1 (red) was compared in MDA-MB-468, MDA-MB-231 cell lines and MCF10A cells stably expressing IQGAP1^IR-WW^ dominant negative with the nuclei stained blue with DAPI. (**B**) Differential expression of centrosome markers in TNBC cell lines: Left, representative immunoblot analyses of endogenous IQGAP1 and centrosome markers in two TNBC cell lines as compared with MCF10A as control. Actin was blotted as loading control. Right, quantification of the expression levels of the markers. Error bars are the Means± s. d for *n =* 3, *p-*value: ^*^ 0.05, ^**^ 0.01, ^***^ 0.001, ns = not significant. (**C**) IQGAP1 is differentially phosphorylated in the TNBC cell lines. Extracts from control MCF10A and TNBC cell lines was blotted for endogenous total IQGAP1 or phospho-serine IQGAP1 (left). The band intensities were quantified by densitometry (right). (**D**) IQGAP1 is required for cell proliferation of the TNBC cell lines: The indicated cells were transfected with shRNA scramble control or IQGAP1-targeted-shRNAs, which knocked down IQGAP1 protein level by ?90% (left). Actin was blotted as loading control. Cell proliferation capacity was measured in scramble control and IQGAP1-shRNA treated cells (right).

We examined localization and expression levels of IQGAP1 and centrosome markers, and tested requirement of IQGAP1 for proliferation in TNBC cell lines (Figure 3). Two different TNBC cell lines with different morphologies and isolated from different racial groups were selected. As shown in Figure 3A, IQGAP1 and BRCA1 co-localized differently in the two cell lines. In the MDA-MB-468 cells (middle panel) endogenous IQGAP1-BRCA1 pair was dispersed into the cytoplasm similar to their pattern in the dominant negative IQGAP1^IR-WW^ cells (upper panel). By contrast in the MDA-MB-231 cells, IQGAP1-BRCA1 co-localized on amplified centrosomes similar to their pattern in the dominant active IQGAP1-F or IQGAP1-C cells (lower panel). Thus, it appears that IQGAP1-BRCA1 differentially impacts centrosome phenotype in the TNBC cell lines, prompting us to examin the expression levels of specific centrosome markers in these cells.

The immunoblot and quantification graph in Figure 3B show that, compared to control, IQGAP1 is slightly increased whereas BRCA1 protein was significantly elevated in both TNBC cell lines. Centrin was elevated in the MDA-MB-231, but more so in the MDA-MB-468. Notably, γ-tubulin was highly elevated in both cell lines, but again more so in the MDA-MB-468 cells. By contrast, the level of α-tubulin did not change from control in the MDA-MB-231, but it was significantly diminished in the MDA-MB-468 cells. Given similar expression level of IQGAP1 in both cell lines, it was curious whether genetic mutations in *iqgap1* gene can explain these differences in centrosome markers. Therefore, we performed targeted Sanger sequencing in the critical functional regions of *iqgap1* gene encoding the IR-WW, the GRD, the critical Ser 1443, NLS and aPI, from genomic DNA isolated from both TNBC cell lines. No genetic mutation was detected.

Next, we examined whether IQGAP1 phosphorylation may be responsible for the differences between the two cell lines. Tyrosine phosphorylation of IQGAP1 was not detected in both cells. However, the blot and graph in Figure 3C show that IQGAP1 was highly serine phosphorylated in the MDA-MB-231 cells compared to control and MDA-MB-468 cells. This differential modification raised a question about requirement of IQGAP1 for proliferation in the two cell lines and was examined by RNAi-mediated knockdown and cell proliferation assay. Figure 3D shows that Knockdown of IQGAP1 reduced cell proliferation in both cell lines, indicating that IQGAP1 function is required for proliferation in the two cell lines, but may employ two distinct mechanisms. Because IQGAP1 modulates the activity of important oncogenic pathways like mTOR-Akt and MAPK, depending on phosphorylation status, we examined the activities of key components of these pathways in the TNBC cell lines.

### Differential activation of stress and proliferation signals in the TNBC cells

**Figure 4:**
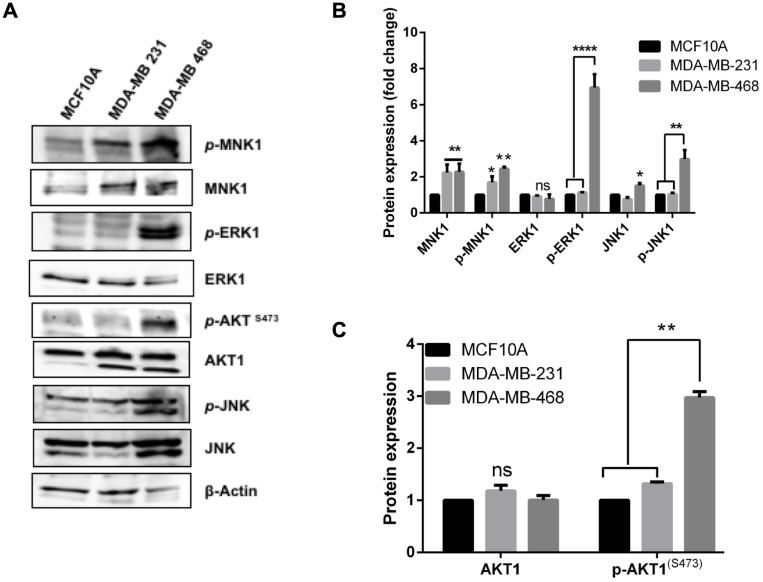
Differential activation of stress and proliferation signals in the TNBC cells. (**A**) Total protein extracts from control MCF10A and the TNBC cell lines was blotted with specific antibodies for total and phospho-form of the indicated kinases to evaluate expression level. Actin was blotted as loading control (**B**) The expression level of total and phosphorylated mitogen-activated protein kinases (MAPK) was quantified by densitometry. (**C**) The expression level of total and phosphorylated AKT was quantified by densitometry. Error bars are the Means± s. d for *n =* 3, *p-*value: ^*^ 0.05, ^**^ 0.01, ^***^ 0.001, ^****^ 0.0001, ns = not significant.

Previously we showed that expression of dominant negative mutants or knockdown of IQGAP1 activates Erk1/2 and JNK stress signal [24, 25] whereas expression of dominant active mutants activated Akt [24, 27]. Figure 4A, 4B shows highly elevated stress signal in the MDA-MB-468 cell lines that display features similar to dominant negative IQGAP1^IR-WW^ cells with respect to centrosome phenotypes like BRCA1 mislocalization (Figure 2). Notably, Erk1/2 was significantly phosphorylated in the MDA-MB-468 as well as Erk1/2 substrate MNK1 along with JNK stress signal. Interestingly, however, Akt1^S473^ activity was also elevated in MDA-MB 468 (Figure 2A, 2C), suggesting that both stress and proliferation signals prevail in this cell line. To this end, the MDA-MB-231 appears to contain active MNK1 only. To identify additional relevant players, we evaluated the expression level and localization-dependent activity of β-catenin, a target of IQGAP1 that is also found in the centrosome.

### Differential expression of β-catenin and IQGAP1 in TNBC cells

**Figure 5:**
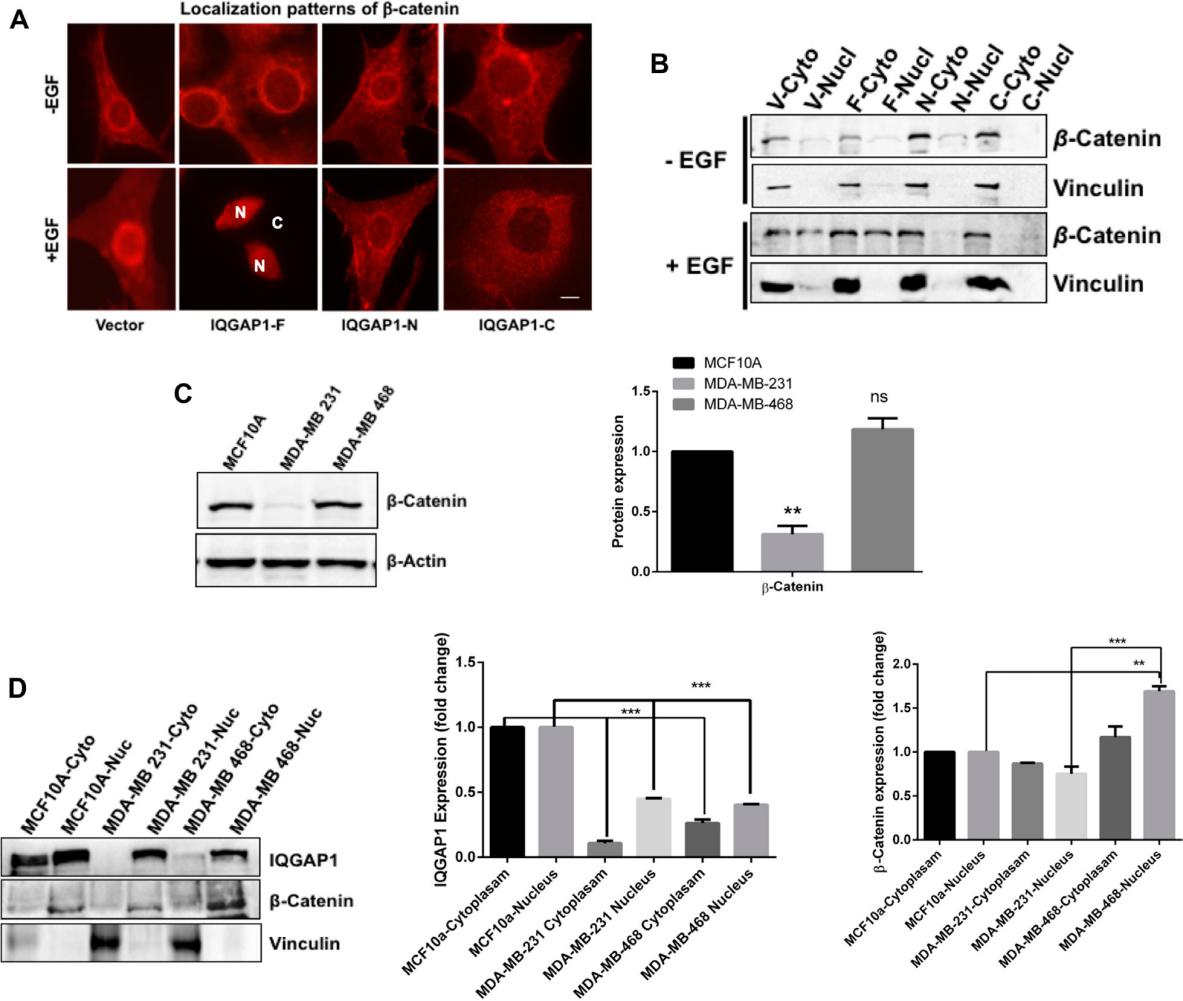
Expression and spatial distribution of IQGAP1 and β-catenin in response to EGF and to cancer. (**A**) IQGAP1 modulates localization of β-catenin: Untreated (upper panels) and EGF-treated (lower panels) MCF10A control cells and stably expressing dominant IQGAP1 constructs, were stained for endogenous β-catenin and visualized by fluorescence microscopy. In the lower panel β-catenin is entirely nuclear in IQGAP1-F cells; N = nucleus; C = cytoplasm. Scale bar = 15 mm. (**B**) IQGAP1 modulates spatial distribution of β-catenin: Upper, representative blot: total proteins from EGF treated and untreated cells expressing IQGAP1 constructs were fractionated biochemically and equal amount of proteins from each fraction blotted with antibodies for β-catenin. Vinculin was blotted as a cytoplasmic fraction control to ascertain clean fractionation. (**C**) Differential expression of β-catenin in TNBC cells: Total proteins from the indicated cancer cells were blotted for β-catenin expression level. Actin was blotted as loading control. Lower graph: β-catenin band intensities were quantified by densitometry: Error bars are the means ± s. d. for *n =* 3 experiments, *p* value ^*^ 0.05, ^**^ 0.01, ns = not significant compared to control (**D**) Stabilization of IQGAP1 and β-catenin in the nuclear fractions of the TNBC cells. Left, total protein extract from control MCF10A and TNBC cell lines was fractionated biochemically and equal amount of the fractions were blotted with antibodies for IQGAP1 (upper panels) and β-catenin (middle panels). Vinculin was blotted to ascertain proper fractionation. Middle graph, IQGAP1 band intensities were quantified by densitometry. Right graph, β-catenin band intensities were quantified by densitometry. Error bars are the Means± s. d for *n =* 3, *p*-value ^*^ 0.05, ^**^ 0.01, ^***^ 0.001.

β-catenin is a component both of centrosomes [45] and of the canonical Wnt signaling pathway where it cooperates with IQGAP1 in controlling adheren junctions [26]. However, whether IQGAP1 impacts β-catenin subcellular localization is unclear. in absence of EGF, β-catenin localized to the cytoplasm and the nuclear envelope in all cells stably-expressing the different IQGAP1 constructs (Figure 5A, upper panels). In presence of EGF, β-catenin translocated to the nucleus only in cells expressing IQGAP1-F. This nuclear translocation did not occur in IQGAP1-N or in IQGAP1-C, suggesting requirement of full length IQGAP1. This finding was further corroborated with biochemical fractionation (Figure 5B). Again, very little β-catenin was found in the nucleus in absence of EGF, however β-catenin translocated to the nucleus in control, but more so in IQGAP1-F cells, thus, substantiating the data in Figure 5A.

Next, the expression level of β-catenin was evaluated in the TNBC cell lines (Figure 5C). Significantly less β-catenin was detected in the MDA-MB-231 cells compared to control and MDA-MB-468 cells. Biochemical fractionation studies supported this finding and further showed that both β-catenin and IQGAP1 are concentrated in the nuclei and are diminished from the cytoplasm of both TNBC cells (Figure 5D). Downregulation of β-catenin in the MDA-MB-231 vs. the MDA-MB-468 cells supports presence of a new mechanism in cancer development. Overall, the data presented above provide tools for cellular and molecular classification of the two TNBC variants, an idea we further examined in primary patient TNBC tumor tissues

### Differential mis-localization of IQGAP1-BRCA1 in human TNBC tumors phenocopies the dominant mutants and the TNBC cells

**Figure 6:**
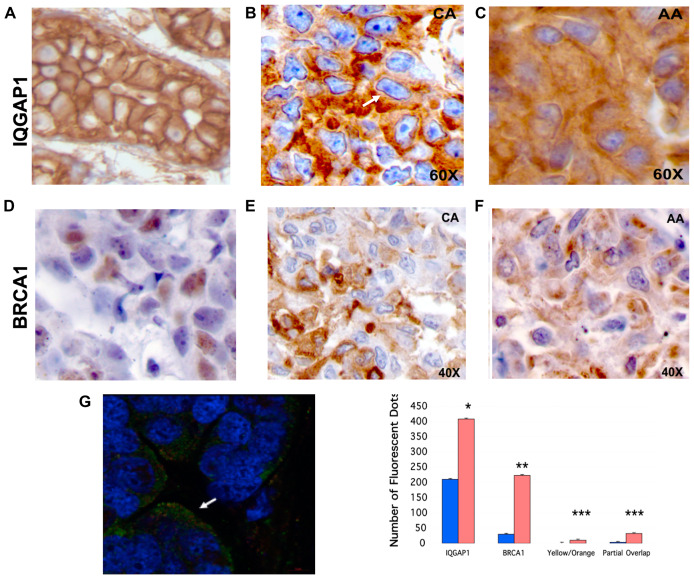
Mislocalization of IQGAP1-BRCA1 in human TNBC tumors phenocopies the dominant mutants and the TNBC cells. (**A**) Normal mammary tissue: chromogen IHC reveals IQGAP1 membranous localization. (**B**) Representative of five individual TNBC primary tumor tissue from Caucasian (CA) patients: chromogen IHC reveals IQGAP1 in the nuclear envelop (arrow) and in cytosolic aggregates. (**C**) Representative of five different TNBC primary tumor tissue from African American (AA) patients: IQGAP1 staining is mainly cytosolic and in aggregates. (**D**) Representative normal mammary tissue: BRCA1 staining is faint and both nuclear and cytosolic. (**E**) Representative of five TNBC primary CA tumor tissue: BRCA1 staining in cytosolic aggregates. (**F**) Representative of five TNBC primary AA tumor tissue: BRCA1 in cytosolic aggregates. (**G**) Quantitative super-resolution analyses of cytosolic IQGAP1 and BRCA1 in TNBC tumors: triple staining of IQGAP1, BRCA1 and the nuclei visualized by super-resolution microscopy (left). IQGAP1 (green) and BRCA1 (red) reside in invasive front (arrow). Quantification of IQGAP1 and BRCA1 in cytosol (orange bars) and in nuclei (blue bars) of TNBC patient tumors (right). Distinct pools of IQGAP1 and BRCA1 are found individually, co-localized (yellow/orange) or partially overlapping (partial overlap). Error bars are the Means± s. d for *n =* 3, *p*-value ^*^ 0.05, ^**^ 0.01, ^***^ 0.001.

To evaluate clinical significance of the above findings, localization of IQGAP1 and BRCA1 was examined in TNBC patient tumor tissues obtained from five African American and five Caucasian women. As with cell lines, two different patterns of IQGAP1 and BRCA1 localization was observed (Figure 6A–6F). In normal tissues, IQGAP1 staining was membranous as expected (Figure 6A). In a pattern observed in the Caucasian samples, IQGAP1 was perinuclear decorating the nuclear envelope similar to that of the dominant active mutants (Figure 2), and was also found in aggregates in the cytoplasm (Figure 6B). A different pattern associated with the samples from African American patients, where IQGAP1 was found in aggregates and/or evenly dispersed throughout the cytoplasm (Figure 6C) similar to the pattern in the dominant negative mutants and the MDA-MB-468 TNBC cells (Figures 2 and 3). In normal tissue, BRCA1 expression was very low and found in both nucleus and cytoplasm (Figure 6D). In Caucasian and African American tumors, BRCA1 was aggregated in the cytoplasm (Figure 6E, 6F).

IQGAP1 and BRCA1 co-localization in the TNBC tumors was also examined by immunofluorescence and super-resolution microscopy (Figure 6G). Distinct pools of the two proteins were observed as individual, co-localized, or partially co-localized. Interestingly, both proteins were found at invasive front in tumor tissues; the significance of which will be investigated in future studies. Overall, these patterns are consistent with the two patterns observed in the cell lines and the dominant mutants and potentially provide tools for classification of the TNBC subtype into molecular variants.

## DISCUSSION

### IQGAP1 can drive centrosome abnormalities

In this study we report a number of novel findings presenting new mechanisms for IQGAP1 in tumorigenesis by modulating centrosome function. Importantly these mechanisms have potential for classifying two distinct variants of TNBC with potential application to other types of cancer. In quiescent cells, IQGAP1 localized to the nuclear envelope and extended into the centrosome (Figure 1C, upper panels). Expression of IQGAP1^IR-WW^ associated with enlarged centrosome and multiple nuclei (Figure 1C, lower panels). This is consistent with our previous finding that IQGAP1^IR-WW^ expression acts as dominant negative that inhibits cytokinesis and produces multiple nuclei [24]. It is uncertain at present whether the enlarged centrosome observed in these cells is a structural or a numerical defect, as several possibilities can account for this phenotype. First, the observed increase could be due to increased PCM seen in other cells [46, 47]. Second, the increased size could result from clustering of amplified centrosomes [48]. It is intuitively appealing that the increased size arose from failure of centrosome division, leading to unipolar centrosome and thus multiple nuclei. Although this idea may require further studies with electron microscopy and additional centriolar markers, it has substantial support. First, centrosome clustering occurs in early mitosis and these cells are in interphase. Second, these mutant cells do not display amplified centrosomes at any stage of the cell cycle, instead they exhibit multiple nuclei hooked to a single enlarged centrosome [24], (Figure 1C). Importantly, this notion is substantiated by the finding that expression of IQGAP1^IR-WW^ in HeLa cells that exhibit amplified centrosomes, produced the same phenotype (see below).

By contrast, expression of dominant active IQGAP1 associated with amplified centrosomes (Figure 1D), supporting the idea that IQGAP1 modulates centrosome division. Further support is that overexpression of dominant negative IQGAP1^IR-WW^ in cancer cells with amplified centrosomes reversed this phenotype and produced enlarged centrosome (Figure 1E). Numerical centrosome aberration has been associated with high-grade tumors and was suggested as a biomarker for advanced cancer [37, 42]. Interestingly, this phenotype was coupled with IQGAP1 localization at the nuclear envelope (Figures 1 and 2). The mechanism by which IQGAP1 expression increases centrosome number is uncertain at present. Ample evidence suggests that centrosome amplification can result from centriole over duplication stemming from overexpression or stabilization of centriolar proteins [49, 50]. Increased PCM components such as γ-tubulin has also been implicated in centrosome amplification [12]. Our results agree with these reports and show that IQGAP1 largely influences and may modulate the expression levels or stability of the different centrosome markers (Figure 1F). While the level of γ-tubulin protein was significantly lower in dominant active IQGAP1-C, the level of acetylated α-tubulin is significantly lower in IQGAP1-F (Figure 1F), indicating that activation of IQGAP1 imbalances these markers one way or the other. By contrast, the centriolar protein, centrin, was significantly down-regulated in dominant negative IQGAP1 cells (Figure 1F), further supporting the hypothesis that aberrant centriolar duplication leads to the enlarged centrosome phenotype as well as the failure of cytokinesis observed in these cells. Interestingly, pericentrin seems to require balanced IQGAP1, as its level was diminished with expression of active or inactive mutants. Overall, these findings present a novel role for IQGAP1 in modulating the levels of centrosome proteins, failure of which leads to centrosome aberrations associated with cancer. Another protein implicated in the stability of centrosome proteins is BRCA1, and here we show that it appears to act through IQGAP1-pathway.

### IQGAP1 influences BRCA1 subcellular distribution

We report that BRCA1 is a novel partner of IQGAP1 and that mislocalization or imbalanced subcellular distribution of BRCA1 and IQGAP1 associates with centrosome aberrations (Figure 2). While, gene deficiency of *brca1* has been associated with amplified centrosomes in breast cancer [12], the data in Figure 2A show that IQGAP1 co-localizes with BRCA1 on amplified centrosomes when dominant active IQGAP1 is overexpressed. Additionally, the two proteins co-localized at interval points on the nuclear envelope in the same cells (Figure 2B, upper panels). Logically, we surmised that these interval locales represent physical interaction at nuclear pores where the two proteins influence centrosome-nuclear crosstalk. In support, expression of dominant negative IQGAP1 abolished this localization pattern where both proteins aggregated in the cytoplasm outside of the nucleus (Figure 2B, lower panels). Furthermore, physical interaction was detected biochemically both ways (Figure 2C). This finding substantiates the notion that IQGAP1 controls centrosome amplification by regulating BRCA1 localization, anchorage and/or transport. This is particularly relevant as ample evidence shows that BRCA1 inhibits centrosome amplification by controlling the stability of centrosome proteins, explaining why BRCA1 depletion leads to supernumerary centrosomes [12–14]. Intuitively, the same effect would occur if active IQGAP1 sequestered BRCA1 in space, as we observe here. Additionally, both IQGAP1 and BRCA1 have been independently implicated in nuclear-cytoplasmic transport where IQGAP1 binds the importin-β5 [51] and BRCA1 binds the importin α subunits [52] of the nuclear pore complex. It is possible that IQGAP1 modulates BRCA1 transport, failure of this process alters BRCA1 localization and thus mimics *BRCA1* depletion, leading to aberrant centrosome phenotypes seen in IQGAP1 dominant mutant cells (Figures 1 and 2). Several lines of compelling evidence supports this idea. First, both proteins have nuclear localization signals and IQGAP1 binding to importin-β5, an effector of Ran GTPase, is required for nuclear pore complex (NPC) assembly and for nuclear-cytoplasm transport [52]. Second, BRCA1 is known to undergo nuclear-cytoplasm transport [53], which has been shown to be essential for centrosome duplication [53, 54]. Hence, IQGAP1-BRCA1 subcellular balance through regulated nuclear-cytoplasm transport would be a novel process in centrosome dynamics, dysfunction of which leads to cancer. This idea was substantiated in cancer cell lines as discussed below

### Differential expression and/or spatial distribution of IQGAP1 and centrosome markers in TNBC cell lines

The results obtained from IQGAP1 mutant analysis were substantiated in TNBC cancer cell lines, using centrosome markers. The two mechanisms associated with dominant mutants of IQGAP1 appear to exist in cancer cell lines (Figure 3). IQGAP1 and BRCA1 were dispersed in the cytoplasm of MDA-MB-468 cells similar to their pattern in dominant negative IQGAP^IR-WW^ cells, but co-localized on amplified centrosomes in MDA-MB-231 cells similar to their pattern in dominant active IQGAP1-C cells (Figure 3A). Enlarged centrosome was not observed in MDA-MB-468, as it likely represents an earlier step in carcinogenesis modified or lost in established cancer cell lines. Expression levels of IQGAP1 and BRCA1 was slightly elevated associating with significantly high levels of centrin and γ-tubulin (Figure 3B), known to associate with centrosome aberrations [12, 49, 50]. Strikingly, the MDA-MB-468 cells displayed significantly diminished levels of acetylated α-tubulin compared to control and to MDA-MB-231 cells (Figure 3B) and similar to cells expressing dominant negative IQGAP1-N (Figure 1F). Therefore, diminished acetylated α-tubulin level may explain arrested centrosome division seen as enlarged size in cells expressing the dominant negative IQGAP1 mutant. The differences in IQGAP1-BRCA1 localization and expression levels may have direct impact on the levels of the centrosome proteins and likely explain the differences in centrosome abnormalities observed in the two cancer cell lines. A major contributing factor, however, appears to be the level of IQGAP1 phosphorylation, as the MDA-MB-231 cells had significantly higher level of *p*Ser-IQGAP1 compared to MDA-MB-468 and control (Figure 3C). Indeed, amplified centrosomes associated both with MDA-MB-231 and dominant active *p*IQGAP1 (Figures 1 and 2). Previously, we showed that expression of active mutants increases IQGAP1 serine phosphorylation and that cycling of IQGAP1 is important to cell homeostasis [27, 44]. In turn, this cycling may be important to balanced level of centrosome proteins and centrosome division. These findings are in line with the results from mutant analysis (Figures 1 and 2), presenting two distinct mechanisms for IQGAP1 in centrosome aberration and cancer. In support of this notion, IQGAP1 was required for proliferation in both cell lines (Figure 3D). Aberrant IQGAP1-centrosome proteins could deregulate key pathways controlled by different domains of IQGAP1 that may be co-opted by cancer cells for oncogenic development, which we find to be the case as discussed below.

### A novel Erk1/2-MNK1-JNK-Akt-β-catenin signature as a new player in IQGAP1-pathway in cancer

The two distinct centrosome phenotypes identified in IQGAP1 mutants and TNBC cell lines appear to coincide with distinct signaling signatures in the TNBC cell lines. These signatures suggest that the normal interplay of active *p*IQGAP1-Akt1 and inactive IQGAP1-Erk1/2 axes that modulate cell proliferation [24, 27, 44] has been rewired in the two TNBC cell lines. Previously, we showed that knockdown of IQGAP1 led to elevated JNK stress signal [25] and that, in response to EGF, IQGAP1 negative mutants activated Erk1/2 signal while IQGAP1 active mutants activated Akt1 [24]. Rewiring of these two distinct pathways appears to occur in one TNBC variant to activate Akt1 alongside Erk1 and JNK stress signal. The MDA-MB-468 cells that resemble IQGAP1 dominant negative cells exhibited significant increases in Erk1/2, JNK and Akt1 activities (Figure 4A–4C). This signal was routed through Mnk1 (Figure 4A, 4B), an effector of Erk1, also known as MKNK1- Mitogen-Activated Protein Kinase (MAPK)-interacting serine/threonine kinase that has been implicated in regulating mRNA translation, oncogenesis, drug resistance and inflammation [55]. The role of Mnk1 in cancer development has been obscure and these findings implicate it, for the first time, in the TNBC subtype downstream of IQGAP1. The MAPK family controls diverse cellular functions, including inflammatory response, differentiation, cell cycle, cell proliferation, gene expression, and apoptosis [56]. Whereas Erk1/2 cascade is activated by mitogens and impacts cell proliferation, the JNK pathway is activated by stress signal and impacts apoptosis [56, 57]. However, rewiring of these two distinct MAPK pathways whereby Erk1/2 up regulates JNK has been reported in cancer [58]. JNK may also be activated via a compensatory proliferation mechanism known as apoptosis-induced proliferation [59] that cancer cells propagate to evade cell death [60]. This mechanism also appears to involve Wnt-β-catenin signaling, which further explains activation of Akt1 in MDA-MB-468 alongside Erk1-JNK (Figures 4 and 5). While it is uncertain how Akt1 is activated in these cells, one can surmise that prevalence of growth factors in MDA-MB-468 cells leads to IQGAP1-mediated Akt1 activation (Figure 4A) and consequent β-catenin translocation to the nucleus (Figure 5, see below). Thus, this observation may represent a rewiring of MAPK and Akt1-β-catenin in cancer. Akt1, a binding-partner of IQGAP1, is involved in regulating the activity of the IQGAP1 adheren junction partner β-catenin that has been implicated in centrosome function [45]

This study reveals that, like IQGAP1, β-catenin also localizes to the nuclear envelope and that IQGAP1 modulates β-catenin transport to the nucleus via different domains in response to growth factor or in cancer (Figure 5A–5D). Expression level of β-catenin was elevated in the MDA-MB-468 cells (Figure 5C) in line with Akt1 activity (Figure 4C), likely mediated by accumulation of IQGAP1 in the nucleus (Figure 5D). Activated Akt binds the Axin-GSK3aβ inhibitory complex, phosphorylates GSK3aβ and thus increases free β-catenin levels [61, 62]. In contrast, the level of β-catenin was significantly diminished in MDA-MB-231 cells, further supporting the existence of two distinct IQGAP1 signaling signatures in these cell lines. Despite differential expression levels of β-catenin in the two cell lines, both IQGAP1 and β-catenin were stabilized in cell nuclei (Figure 5D) similar to EGF treated cells (Figure 5B), perhaps hinting to an IQGAP1-mediated activation of EGFR particularly in MDA-MB-468 cells. This also is consistent with the finding that IQGAP1 binds the importin-β5 subunit of the nuclear pore receptor and facilitates shuttling of β-catenin to the nucleus [51]. Thus an IQGAP1-EGFR deregulation in addition to Akt1 activation may be responsible for concentrating IQGAP1-β-catenin in the nucleus (Figures 4, 5D). Nuclear β-catenin is a known co-activator of gene expression via LEF/TCF retention [63]. As discussed earlier, mounting evidence supports that cytoplasmic-nuclear transport as well as the nuclear pore complex proteins are essential for cell abscission, centrosome duplication and invasive cancer phenotype [53, 54, 64, 65]. Indeed, β-catenin is a component of centrosomes [45] and plays a role in centrosome amplification [66] where it has been associated with human cancers [67]. Taken together, these findings suggest a novel shared role for IQGAP1-BRCA1 in cytoplasmic-nuclear transport and centrosome duplication leading to cancer development and more research is underway to further examine this notion. These events may operate downstream of IQGAP1-MAPK axis, hijacked differentially by cancer cells. This hijack manifests in one pathway existing in MDA-MB-468 involving IQGAP1-Erk1-Mnk1-Akt1-β-catenin and another existing in MDA-MB-231 involving *p*IQGAP1-Mnk1 both differentially influencing centrosome division and leading to cell proliferation. As these findings can provide molecular basis for personalized medicine, they were substantiated in clinical samples

### Differential localization of IQGAP1 and BRCA1 in patients’ tumors phenocopies the mutants

Clinical significance was established, as patient TNBC tumors displayed distinct phenotypes relative to IQGAP1 and BRCA1 localization (Figure 6), resembling the IQGAP1 mutants and the TNBC cell lines. The normal membranous localization of IQGAP1 (Figure 6A) became mostly perinuclear decorating grooved nuclei in a set of tumors obtained from Caucasian patients (Figure 6B). This phenotype resembles IQGAP1 nuclear envelope localization in the active mutants (Figure 2). Importantly, grooved nuclei phenotype is hallmark of highly invasive tumors known to result from dysfunction of nuclear envelope proteins [65, 68, 69], thus further substantiating the oncogenic role of IQGAP1. This phenotype was diminished or absent from the set of tumors isolated from African American patients (Figure 6C), resembling localization in IQGAP1 negative mutants (Figure 2). However, the two tumor types shared aggregated IQGAP1 in the cytosol (Figure 6B, 6C). While BRCA1 was found in the nuclei and the cytosol of normal tissues, confirming previous reports [70, 71], the cytosolic ratio increased in both tissues and resided in aggregates overlapping IQGAP1 (Figure 6E–6G). Quantitative super-resolution microscopy clearly confirmed the cytosolic shift and uncovered interesting patterns of IQGAP1-BRCA1 partial or complete overlap (Figure 6G), indicating shared and distinct functions of the two proteins. While the nature of the observed aggregates is to be investigated, protein aggregation is recognized as hallmark of cancer linked to endoplasmic reticulum (ER) stress [72, 73]. It may also represent malfunctioned lysosomes also linked to cancer and ER stress [74, 75]. Aberrant activation of signaling proteins like mTORC1 a key IQGAP1 partner in insulin secretion and cell size, and E-cadherin, an IQGAP1 partner in cell adhesion, by lysosome targeting has been reported [76, 77]. Overall, these localization patterns establish two distinct molecular signatures in agreement with the findings from *in vitro* mutant analysis and TNBC cell line studies. These molecular signatures have potential in classification of the heterogenous TNBC and could provide highly sought-after therapeutic targets. It is tempting to assume that these differences indicate measurable tools for cancer racial disparity, however an expanded study with a large sample of tissues will be required before firmly arriving to such conclusion.

**Figure 7:**
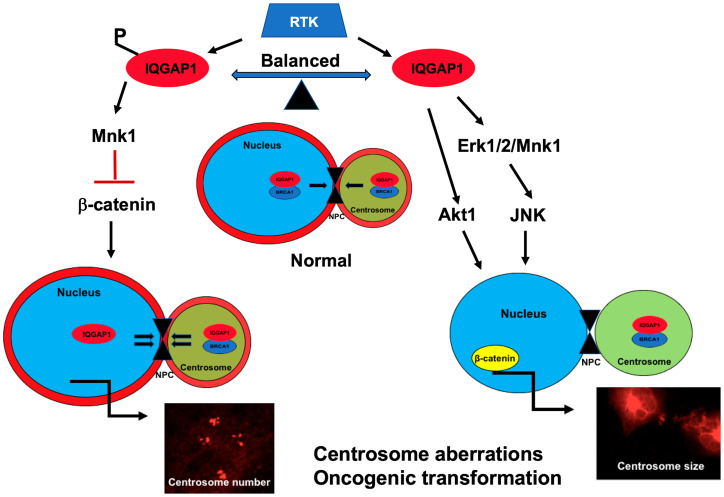
A Model of IQGAP1 role in centrosome-nuclear crosstalk in cancer: IQGAP1 is a phosphorylation-dependent regulatory scaffold that modulates shuttling of centrosome proteins like BRCA1, acting downstream of receptor tyrosine kinase (RTK) such as EGFR. Balanced phosphorylation-dephosphorylation cycling and dynamic localization normally couples centrosome size and division. Right: chronically unphosphorylated IQGAP1(dominant negative), activates a novel rewired Erk-Mnk1-JNK-Akt1-β-catenin signaling pathway and displaces IQGAP1 (red) from the nuclear envelope, thereby diminishing centrosome-nuclear transport- via nuclear pore complex (NPC)- known to be required for centrosome division [54], leading to enlarged centrosome. These events alter BRCA1 centrosome-nuclear distribution, causing cytosolic aggregates seen in certain variants of TNBC and other cancers. Left: Chronically phosphorylated IQGAP1 (dominant active) activates Mnk1, suppresses β-catenin expression and nuclear translocation, enhances centrosome-nuclear transport, thereby leading to centrosome amplification seen in certain variants of TNBC and other cancers. Accordingly, IQGAP1 generates cancers by, at least, two context-dependent pathways and can serve as a common therapeutic target or biomarker in heterogenous cancers like TNBC.

Taken together, the findings of this study underscore the importance of the delicate balance of expression, localization and/or modification of IQGAP1-BRCA1 and centrosome proteins in cell homeostasis and support that IQGAP1 influences BRCA1 transport or anchorage. IQGAP1 may serve as a regulatory scaffold for BRCA1 and other centrosomal proteins to regulate their stability or transport between the nucleus and the centrosome, a mechanism by which it modulates nuclear-centrosome crosstalk during the cell cycle and thus regulates cell proliferation (Figure 7). Furthermore, as IQGAP1 has been implicated in various carcinomas, the mechanisms discussed here likely apply to a wide range of carcinoma, thus presenting IQGAP1 as a non-organ-specific clinical target amenable to precision medicine

## MATERIALS AND METHODS

### Cell culture and chemicals

Human triple negative breast cancer (TNBC) cells MDA-MB-468 from an African American patient and MDA-MB-231 from a Caucasian patient, MCF10A-immortalized non-tumorigenic “normal” mammary epithelial cells and human cervical cancer HeLa cells were purchased from ATCC. The cells were grown per the manufacturer’s instructions in DMEM (MEM for HeLa, MEGM for MCF10A), containing 100-units/ml penicillin, 100 μg/ml streptomycin (Invitrogen) supplemented with 10% fetal bovine serum (FBS). Cells were maintained in a humidified incubator at 37°C and 5% CO_2_. The V5-IQGAP1 constructs and generation of stable cell lines were previously described [27, 24]. All chemicals were molecular biology grade obtained from Fisher or Sigma-Aldrich.

### Antibodies

All antibodies were obtained from reliable biotech companies and validated by recognition of control vs. total proteins isolated from knockout animals, RNAi-mediated knockdown cells or tagged proteins. Monoclonal antibodies for IQGAP1 rose in rabbit or mouse were previously described [24] and were obtained from Pierce (Thermo-Fisher Scientific) or Santa Cruz Biotechnology (Santa Cruz, CA). Monoclonal antibodies against V5 were from Invitrogen. Monoclonal antibodies for BRCA1 raised in rabbit or mouse were obtained from Pierce and Santa Cruz Biotechnology (Dallas, TX). Antibodies against centrin, pericentrin, α- and γ-tubulin, PKC-substrate pan phosphoserine were purchased from Cell Signaling Technology (Beverly, MA).

### Sub-cellular fractionation assay

Nuclear and cytosolic fractionation was performed essentially as described previously [78]. Briefly, ~80% confluent cells were washed with cold PBS, then lysed on ice in a lysis buffer (20 mM HEPES, pH 7.2, 10 mM KCl, 2 mM MgCl_2_, 0.5% Nonidet P40, 1 mM Na_3_VO_4_, 1 mM phenylmethylsulfonyl fluoride, 0.15 units ml^-^ aprotinin or a protease inhibitor cocktail from Fisher), scraped on ice and transferred into pre-chilled microfuges. The lysate was centrifuged at 1,500 × *g* for 5 min to sediment cell debris. The supernatant was then centrifuged at 15,000 × *g* for 10 min at 4° C, and the resulting supernatant was saved as cytosolic fraction. The nuclear pellet was washed three times with lysis buffer and resuspended in same buffer supplemented with 0.5 M NaCl to extract the nuclear proteins. The extracts were centrifuged at 15,000 × *g* for 10 min, and the resulting supernatant was saved as nuclear fraction. A fractionation kit from Thermo Fisher was also utilized with same results. PARP, as a nuclear fraction marker, or vinculin were used to ascertain proper fractionation. Equal amounts of proteins were evaluated by Western blotting as described below, quantified by densitometry, using a Bio-Rad chemiDoc Imager and expressed as histograms, using Microsoft Excel or Graph Pad Prism 6.0.

### Super-resolution confocal microscopy

Cells were cultured in multiple-chamber slides (Nalge, Nunc), washed with PBS and fixed in –20° C methanol for 10 min, permeabilized in PBS containing 1% Triton X-100 and blocked with 1 mg/ml BSA in PBS, incubated with primary or control antibodies overnight at 4° C followed by secondary (Texas Red, Alexa Fluor 555, or Alexa Fluor 488, Molecular Probe) for 1 hr. each at room temperature and the nuclei were stained with DAPI (Sigma or Invitrogen). The centrosome was visualized with centrin, pericentrin, γ-tubulin antibodies, FITC-α-tubulin antibody (Sigma) or monoclonal -α-tubulin from Cell Signaling. The cells were imaged with a Leica confocal microscope or a Zeiss LSM800 Laser Scanning Confocal Microscope with Airyscan. Regular fluorescence was also done with an Olympus fluorescence microscope fitted with Hamamatsu ORCAER monochrome CCD camera and the images were composited in Adobe Photoshop and quantified as described below.

### Genomic DNA purification and sequencing

A genomic DNA purification kit (Thermo Scientific) was used to purify genomic DNA from log phase normal and cancer mammary cell lines. Sanger sequencing of *iqgap1* gene was performed by GENEWIZ, using primers designed to be specific for *iqgap1* gene.

### Fluorescence and chromogen immunohistochemistry (IHC)

De-identified Formalin-fixed paraffin-embedded (FFPE) TNBC tumors and matched normal tissue blocks were obtained from the Tissue Bank of the Brown University Rhode Island Hospital and were certified by the attending pathologist Dr. Evgeny Yakirevich. All procedures were carried out according to the institutional human subject protection and IRB protocols. Consecutive sections from the FFPE tissue blocks were cut at 4 μm, deparaffinized with xylene and rehydrated with graded alcohols.

Chromogen IHC was performed, using an automated station. Microwave antigen retrieval was performed in Dako Target Retrieval Solution (EDTA pH9) for 10 min, followed by cooling for 20 minutes at room temperature. IHC staining was performed with EnVision Dual Link System-HRP with DAB (DAKO, Santa Clara, CA) after blocking with Dako Dual Endogenous Enzyme Block for 10 minutes. Monoclonal antibodies against IQGAP1were used at 1:250 dilutions. Monoclonal antibodies against BRCA1 were used at 1:50 dilution. Appropriate positive and negative controls and H&E were stained in parallel. Immunoreactivity was assessed based on a combined score of the extent and intensity of staining.

For fluorescence IHC, antigen retrieval was done with10 minutes incubation in 10mM sodium citrate pH 6.0 at 85° C. The slides were washed in 1× PBS and incubated in blocking buffer (1% horse serum in PBS) for 30 minutes at room temperature before applying primary monoclonal antibodies against IQGAP1 and BRCA1 at 1:200 dilutions for 1 hr. at room temperature or overnight at 4° C. After extensive washing with the blocking buffer followed by PBS, secondary antibodies, Alexa Fluor 448 and Alexa Fluor 555, were applied at 1:500 dilutions for 1 hr. at room temperature followed by extensive washing and addition of a diluted DAPI solution for 5 minutes at room temperature. The slides were washed again, rinsed with pure water and air-dried before mounting and sealing with clear nail polish. Quantification was done typically from 50-100 cells or from 5 tissue fields chosen at random, using the Zeiss Zen software or the NIH ImageJ. Images were also counted by blinded users.

### Immunoprecipitation (IP)

Cells growing at ~80% confluency were rinsed with ice-cold PBS and scraped into ice-cold NP40 lysis buffer [20 mmol/L Tris (pH 8.0), 137 mmol/L NaCl, 1% NP40, 10% glycerol] supplemented with protease inhibitors (1 mmol/L phenylmethylsulfoxide, 10 μg/mL aprotinin, 10 μg/mL leupeptin) and 3 mmol/L Na3VO4. The lysates were cleared by centrifugation and protein concentration was determined by bicinchoninic acid assay (Pierce, Rockford, IL). Four hundred to 1,000 μL of lysates were pre-cleared with 15 μl of PBS-equilibrated protein G or A beads for 1 hr. and used for immuno precipitation reaction with specific antibodies at 4° C overnight with back-to-back rotation followed by incubation with protein-A/G-Sepharose for 2 hours at 4° C to collect the immune complexes. The beads were washed with NP40 buffer five times, and analyzed by SDS-PAGE and Western blotting as described below.

### Western blotting

The beads containing the immuno-precipitates alongside 30 mg of whole cell lysate (WCL), representing the input, were resuspended in SDS-PAGE sample buffer, boiled, resolved on gradient SDS-PAGE, and transferred into PVD nylon membrane. After blocking, the membranes were blotted with primary antibodies in TBST (50 mmol/L Tris (pH 7.4), 150 mmol/L NaCl, 0.05% Tween 20) plus 1% BSA at 4° C overnight. Following several washes with TBST, the membranes were incubated with horseradish peroxidase-conjugated appropriate secondary antibodies. The specific signals were obtained using the Amersham enhanced chemiluminescent detection system (Arlington Heights, IL) or Super Signal chemiluminescent solution (Thermo-Fisher Scientific) and captured with a Bio-Rad ChemiDoc imager.

### RNA interference (RNAi)

A set of three shRNAs against IQGAP1 and controls (Santa Cruz Biotech), were used following the manufacturer’s protocol as previously described [24, 27, 45]. After 48 h, the cells were counted for proliferation or lysed and protein depletion was evaluated by immunoblotting as described above.

### Statistical analyses

Data are representative of at least three independent experiments with 2–3 replicas each. Statistical analyses were performed using Graph Pad Prism 6.0 (Graph Pad Software, Inc., La Jolla, CA, USA) and SPSS version 17.0 software (SPSS Inc., Chicago, IL, USA). Student’s *t* test, ANOVA and the algorithms in Microsoft Excel software were also used to compare levels between different groups. All statistical tests were two-sided, and *p* values less than 0.05 were considered statistically significant. Fluorescence was quantified from randomly chosen 50 cells or five random tissue fields, using the free online NIH-Image J or the Zeiss Zen Software.

## Abbreviations

TNBC: Triple negative breast cancer
BRCA1: Breast cancer-associated 1
IQGAP1: IQ-containing GTPase Activating Protein
PCM: Pericentriolar material
MAPK: Mitogen-Activated Protein Kinase
WCL: whole cell lysate
CHD: *c*alponin *h*omology *d*omain
GRD: *R*asGTPase-activating protein-*r*elated *d*omain
RGCT: *R*as*G*AP-*C t*erminus
NLS: nuclear localization signal
IQGAP1-F: IQGAP-1 full-length
IQGAP1-N: IQGAP1-N-terminal domain
IQGAP1-C: IQGAP1-C-terminal domain
IQGAP1^IR-WW^: IQGAP1-IR-WW fragment
RTK: receptor tyrosine kinases
NPC: nuclear pore complex
DAPI: 4′,6-diamidino-2-phenylindole
EGF: epidermal growth factor
EGFR: epidermal growth factor receptor
IHC: immunohistochemistry
PBS: phosphate buffered saline
WCL: whole cell lysate
ER: endoplasmic reticulum
